# Relevance between Helicobacter *pylori* Infection and Non-Alcoholic Fatty Liver Disease in Birjand, Iran

**DOI:** 10.25122/jml-2019-0012

**Published:** 2019

**Authors:** Mahyar Mohammadifard, Zeinab Saremi, Mahboobe Rastgoo, Ehsan Akbari

**Affiliations:** 1. Department of Radiology, School of Medicine, Birjand University of Medical Sciences, Birjand, Iran; 2. Department of Internal Medicine, School of Medicine, Birjand University of Medical Science, Birjand, Iran; 3. General Practitioner, Imam Reza Hospital, Birjand, Iran

**Keywords:** Non-alcoholic fatty liver disease, Helicobacter pylori, critical proportion

## Abstract

There is evidence that infection by *H. pylori* can have a critical proportion in the development of hepatocyte injury and both noncancerous and malignant liver conditions including non-alcoholic fatty liver disease (NAFLD). This is attributed to several mechanisms, the most important one being the toxic products of the bacterium *H. pylori* and oxidative injury for hepatocytes which promotes hepatic injury. The present research was aimed at determining the association between *H. pylori* infection and the prevalence of NAFLD in Birjand, Iran. Two groups were included in this cross-sectional study at the outpatient university clinic. One group had NAFLD (65 patients) and the other group was healthy controls without NAFLD (65 subjects). The diagnosis of NAFLD was performed using abdominal ultrasound examination and the absence of taking steatogenic medications or alcohol. Serum anti-*H. pylori* IgG and fecal *H. pylori* antigen were tested for diagnosing of *H. pylori* infection using ELISA method. *H. pylori* infection diagnosis was made if both tests were positive. None of the subjects in either group had symptoms related to the digestive system including dyspepsia, GERD (gastroesophageal reflux disease), or epigastric pain suspicious of peptic ulcer disease. There were 37 patients (28.5%) in both NAFLD (22 cases, 33.8%) and control (15 cases, 23.1%) groups whose *H. pylori* tests (both IgG and fecal antigen) were positive. Statistically, no significant difference was observed between the two studied groups regarding *H. pylori* infection frequency (p = 0.37). Asymptomatic *H. pylori* infection rate was not significantly different between NAFLD patients and control subjects in Birjand, Iran.

## Introduction

Non-alcoholic fatty liver disease (NAFLD) is described as hepatic steatosis (storage of extra fat in hepatocytes) in those who do not consume significant amounts of alcohol. Although this definition is general, the spectrum of the condition ranges from a simple fat accumulation to a more advanced disease characterized by steatohepatitis and inflammation. Its prevalence is increasing worldwide and it is considered the most common hepatic condition in Western countries [1]. The most important risk factors related to NAFLD are central obesity, diabetes type 2, dyslipidemia, and mainly metabolic syndrome. In fact, NAFLD is regarded as the hepatic manifestation of the metabolic syndrome[2].

The exact biological mechanism of NAFLD is not fully clarified, but insulin resistance has been proposed as the main pathogenesis factor in NAFLD [3]. But, as NAFLD is considered a complex disease, several other genetic and environmental factors have been implicated in NAFLD pathogenesis [4]. For example, a “second hit” hypothesis or additional oxidative injury mechanism has been described. In other words, the “first hit” is fat accumulation in the hepatocytes and the “second hit” is an oxidative injury which promotes hepatic injury [5]. Factors that have been described to have a role as the second hit are lipid peroxidation, release of toxic products, and the like. One of the factors recently noted in the pathogenesis of NAFLD as the second hit is gastrointestinal tract microbes or bacterial overgrowth [6, 7]. It is assumed that these microbes release toxic products which can reach the liver via the portal vein [8, 9]. One of these microbes commonly found in humans’ stomach is *Helicobacter pylori* (*H. pylori*). *H. pylori* are a type of Gram negative, microaerophilic bacteria. The size of this the microorganism is about 3.5 microns in length and 0.5 microns in width. *H. pylori* survive in the gastric epithelium. This is due to its urease, which hydrolyzes gastric urea, spiral shape, which allows the organism to pass through mucous layers, flagella, and ability to adhere to the gastric epithelium. Infection caused by *H. pylori* is recognized as the most prevalent chronic bacterial infection in humans. *H. pylori* infections in childhood are more common in developing countries. Its prevalence among adults exceeds 80% in developing countries and about 30% in developed nations [10].

The observations regarding the possible function of *H. pylori* in NAFLD and other liver diseases have been discussed recently. In one study, *H. pylori* was reported as a non-dependent risk factor for NAFLD [11] and in another study, its DNA was detected in a female patient with non-alcoholic steatohepatitis (NASH) [12]. Then, in a study on 28 biopsy-proven NAFLD patients and 25 healthy controls, it was reported that 82% of NAFLD group vs. 56% of healthy controls had anti-*H. pylori* IgG seropositivity. The authors concluded that *H. pylori* is another factor that should be considered in the “second hit” hypothesis [7]. On the other hand, some studies showed contradictory results indicating that *H. pylori* is not related to NADLD using hepatic steatosis index [13] or using regression models to demonstrate independent risk factors for fatty liver disease (FLD) or NAFLD [14, 15].

As described above, regarding the existence of controversy about the probable role of *H. pylori* infection in NAFLD and limited studies, we decided to conduct the current study to elucidate the association between *H. pylori* infection and NAFLD. As both NAFLD and infection by *H. pylori* are common in the general population and the fact that effective eradication treatments are available for *H. pylori*, we think that the results of this study would strengthen the current knowledge about this discipline.

## Materials and Methods

### Population study, sampling and research design

In this cross-sectional study, the study population consisted of patients who presented to the Outpatient Gastroenterology Clinic of our university hospital in 2014. The patients were referred to our referral center for making a diagnosis of NAFLD and providing appropriate management. Liver ultrasound was performed by a board-certified radiologist to diagnose NAFLD. The diagnosis of NAFLD was made using abdominal ultrasound examination results and lack of history of taking medications with potential for hepatic injury and not consuming alcohol per guidelines of the American College of Gastroenterology [16]. The patients were sampled by convenience method. The sample size considering the results of a previous study [7] was calculated as 64 patients in each group. One group had NAFLD based on ultrasound examination (65 patients) and the other group was controls without NAFLD (65 individuals). The controls were matched regarding age and gender. The control groups were employed from patients who presented for getting regular check-ups. The age range of 18-45 years was the inclusion criteria. Exclusion criteria were taking medications that are used for management of NAFLD (vitamin E, metformin, thiazolidinediones, ursodeoxycholic acid, or herbal medicines/supplements), previous history of *H. pylori* eradication treatment, liver cirrhosis, autoimmune hepatitis, viral hepatitis, primary biliary cirrhosis (PBC), primary sclerosing cholangitis (PSC), Wilson’s disease, taking antibiotics such as amoxicillin, metronidazole, clarithromycin, or ciprofloxacin in the last 6 months, and taking medications that result in hepatic fat deposition (amiodarone, anti-convulsive medications, corticosteroids, and estrogen preparations). None of the subjects in either group had symptoms related to the digestive system including dyspepsia, GERD (gastroesophageal reflux disease), or epigastric pain suspicious of peptic ulcer disease.

### Data collection

For detecting the infection of *H. pylori* i, venous blood sample was obtained. Both, the fecal *H. pylori* antigen and the serum anti-*H. pylori* immunoglobulin G (IgG), were measured by enzyme-linked immunosorbent assay (ELISA) method (Generic Assay, Germany). If the results of both tests were positive, the patients were considered to be *H. pylori* infected. Plus, other laboratory markers including hepatic enzymes (alanine transaminase (ALT) and aspartate transaminase (AST) and lipid profile [HDL (high-density lipoprotein), LDL (low-density lipoprotein), total cholesterol, and triglyceride] were assayed.

### Statistical analysis

The descriptive indices (frequency, percentage, mean, and standard deviation) of the gathered data were expressed by SPSS software for Windows (ver. 21.0). For comparison of *H. pylori* infection rates and IgG antibody titer between the two studied groups, the chi-squared and the student t-test were applied, respectively. (The *p*-value was considered as 0.05).

### Ethics

The study protocol was approved by the Ethics Committee of our medical university. The objectives and details of the study were explained prior to patients’ participation and written informed consent was obtained after their agreement.

## Results

There were 38 females in the NAFLD group (58.5%) and 30 females in control group (46.2%); p = 0.16. Table 1 presents age, gender, and hepatic transaminases comparisons between the two studied groups. In NAFLD group, 33 patients (50.8%) had grade I disease, 30 cases (46.2%) grade II, and two cases (3.1%) had grade III disease.

**Table 1:** Comparison of gender, age, and hepatic transaminases between non-alcoholic fatty liver disease (NAFLD) patients and healthy control subjects

	NAFLD (N = 65)	Healthy control (N = 65)	p value
Age	37.6 (±5.6)	36.6 (±6.1)	0.1
Gender			
Male	27 (41.5%)	35 (53.8%)	0.1
Female	38 (58.5%)	30 (46.2%)	
AST, U/L	26.09 (±11.2)	23.4 (±12.2)	0.2
ALT, U/L	37.3 (±21.3)	27.06 (±24.6)	0.01

The serum level of anti-*H. pylori* IgG was positive in 38 patients of NAFLD group (58.5%) and in 37 subjects of control group (56.9%); p = 0.86. Mean (±SD) of anti-*H. pylori* IgG in NAFLD group (78.1 ± 9.9 IU/mL) was more than control group (51.7± 7.2 IU/mL); p = 0.03. Positive fecal *H. pylori* antigen test was also more frequent in NAFLD group compared to the control group (26 patients (40%) vs. 18 subjects (27.7%)), but the difference was not statistically significant (p = 0.13). The prevalence of *H. pylori* infection was greater when serum IgG test was considered rather than the fecal antigen test result in both NAFLD and control groups.

Table 2 shows a comparison of mean (±SD) values of anti-*H. pylori* IgG levels between the two groups of patients with and without positive fecal *H. pylori* antigen test in both NAFLD and control groups. As seen, the mean values of anti-*H. pylori* IgG titers were notably higher in those with a positive fecal antigen test. Both low and high levels of *H. pylori* IgG titers were seen in tested groups. It was not possible to define a cut-off value for *H. pylori* IgG to predict those with positive or negative fecal antigen test.

**Table 2:** Comparison of mean (SD) values of anti-*H. pylori* IgG titers between two groups of patients with and without positive fecal *H. pylori* antigen test

	Total	Mean (±SD) *H. pylori* IgG titer	Range of *H. pylori* IgG titer	p value
Positive fecal antigen test, IU/mL	44	96.9 (±73.8)	6-289	< 0.001
Negative fecal antigen test, IU/mL	86	48.6 (±64.4)	9-261	

There were 37 patients (28.5%) in both NAFLD and control groups whose *H. pylori* tests (both IgG and fecal antigen) were positive. In Table 3, the distribution of patients with at least one and both *H. pylori* positive tests are presented. As seen, no significant difference was observed between the studied groups regarding the frequency of *H. pylori* infection considering both positive *H. pylori* IgG and fecal antigen test results.

**Table 3:** Distribution of patients with both positive *H. pylori* tests (anti-*H. pylori* IgG and fecal antigen test) and at least one positive test in NAFLD and control groups

	Total	NAFLD (N= 65)	Control (N = 65)	P value
Both tests were positive	37 (28.5%)	22 (33.8%)	15 (23.1%)	0.17
At least one test was positive	82 (63.1%)	42 (64.6%)	40 (61.5%)	0.71

Table 4 presents a comparison of lipid profile between NAFLD patients and healthy control subjects.

**Table 4:** Comparison of lipid profile between non-alcoholic fatty liver disease (NAFLD) patients and healthy control subjects

	NAFLD (N = 65)	Healthy control (N = 65)	p value
Total cholesterol, mg/dL	196.2 (±37.04)	177.9 (±43.2)	0.01
HDL, mg/dL	44.5 (±16.3)	42.1 (±9.4)	0.3
LDL, mg/dL	120.4 (±34.5)	116.3 (±35.2)	0.5
Triglyceride, mg/dL	169.6 (±81.3)	121.9 (±43.7)	< 0.001

All data are presented as mean (standard deviation)

HDL= high-density lipoprotein; LDL= low-density lipoprotein

## Discussion

According to the current findings, we did not observe any significant difference concerning *H. pylori* infection rate between NAFLD and healthy controls. As both *H. pylori* infection and NAFLD are usual health conditions, attempts have been accomplished to investigate the possible function of *H. pylori* in NAFLD pathogenesis. These studies have been done based on the reports that *H. pylori* bacterium has been implicated in some extra-digestive diseases such as cardiovascular, neurologic, and hepatobiliary conditions [17, 18]. The association, not a causative link, between *H. pylori* and insulin resistance has been reported [19, 20] and since insulin resistance is a major pathogenesis factor in NAFLD, attention has been made regarding the probable role of *H. pylori* in liver diseases, especially NAFLD. Studies about the extra-gastric role of *H. pylori* in hepatobiliary system have not been limited just to NAFLD. Even in hepatocellular carcinoma and cirrhosis, *H. pylori* DNA has been detected in a considerable number of liver biopsy samples [21]. In a previous study recruiting 28 NAFLD cases (15 with NAFLD and 13 with NASH) and 25 controls, the authors found that a higher percentage of NAFLD patients (23 out of 25, 82.1%) was seropositive for anti-*H. pylori* IgG than control group (14 out of 25, 56%); p = 0.03. [7] Here, we excluded those who had received *H. pylori* eradication treatment in their life or those who had consumed antibiotics implicated in eradiation treatments in the preceding six months. Also, we did not recruit those with NASH in the current study. However, in the mentioned study [7] one subject in control group and six patients in the NAFLD group had a history of receiving *H. pylori* eradication treatment. When the authors combined *H. pylori* seropositivity with *H. pylori* eradication treatment, the difference between these groups became even more significant (92% in NAFLD vs. 56% in control; p = 0.002). When they conducted more analyses in the subgroups of NAFLD and NASH, incorporating a history of receiving *H. pylori* eradication therapy, urea breath test, and IgG, no difference was seen between NAFLD and NASH subgroups [7]. The authors concluded that *H. pylori* could represent a contributing function in the development of NAFLD, but does not lead to progression from NAFLD to NASH [7]. Here, we used both IgG and fecal antigen tests as objective evidence for *H. pylori* infection diagnosing. In a previous study in Iran [22], fecal antigen test was found to be a non-invasive test with 87.8% sensitivity and 75% specificity. But serum IgG had only 50% sensitivity. Based on this report, requesting for fecal antigen test would be better assay for diagnosis of *H. pylori* infection.

In contrast to the above-mentioned study, two studies did not find any relation between *H. pylori* and NAFLD. In a large study on 13,737 subjects in Japan anti-*H. pylori* antibody was assayed to diagnose *H. pylori* infection, and abdominal ultrasound was used to make the diagnosis of FLD and NAFLD. By conducting a multivariate regression analysis, the authors reported that *H. pylori* infection was not in connection with NAFLD in either gender (p values of 0.7 in females and 0.4 in males [14]. In another study in South Korea, 4030 patients were studied using urea breath test for diagnosis of *H. pylori* and NAFLD was according to the hepatic steatosis index and NAFLD liver fat score [13]. Similar to the other study from Japan, the authors did not find *H. pylori* infection as a significant risk factor for NAFLD based on regression analysis.

The studies regarding this topic have also tried to show the role of *H. pylori* eradication on liver fat content and its function. In a randomized clinical trial, Jamali et al. [23] applied *H. pylori* IgG to diagnose the resulting infection and urea breath test for determining the eradication of *H. pylori* six weeks after administration of standard *H. pylori* eradication treatment on 100 dyspeptic patients with increased aminotransferase levels. They found that *H. pylori* eradication did not have a significant effect on the function and fat content of hepatic.

Although a greater *H. pylori* IgG titer was seen in NAFLD patients compared to the control groups, combined IgG and fecal antigen tests showed no significant difference in diagnosis of *H. pylori*. Even when one of these tests was considered positive, no significant difference was seen between the two studied groups. Here, the patients included were asymptomatic regarding digestive system complaints. We think that maybe in further studies recruiting two groups of patients, one with symptomatic positive *H. pylori* infection and the other being an asymptomatic sample with positive *H. pylori* infection, and comparing these two regarding the relationship between *H. pylori* infection and NAFLD will add to the current knowledge. In other words, maybe differences in liver fat content exist in those who have symptomatic *H. pylori* infection.

In light of the observed results and the body of evidence in the literature, it seems that *H. pylori* infection might not have clinical significance in NAFLD patients. When it hypothesized several years ago that *H. pylori* eradication can be functional in the management of NAFLD, it was so promising to study this topic. However, at the current time with the evidence exists, it is suggested to consider other established treatment options and focus further studies on other oxidative injuries.

## Conflict of Interest

The authors confirm that there are no conflicts of interest.
